# CXCL13 Promotes Proliferation of Mesangial Cells by Combination with CXCR5 in SLE

**DOI:** 10.1155/2016/2063985

**Published:** 2016-09-08

**Authors:** Zhanyun Da, Liuxia Li, Jin Zhu, Zhifeng Gu, Bo You, Ying Shan, Si Shi

**Affiliations:** ^1^Department of Rheumatology, Affiliated Hospital of Nantong University, Nantong, Jiangsu Province, China; ^2^Department of Otorhinolaryngology Head and Neck Surgery, Affiliated Hospital of Nantong University, Nantong, Jiangsu Province, China

## Abstract

As a CXC subtype member of the chemokine superfamily, CXCL13 is considered to be involved in systemic lupus erythematosus (SLE), especially in lupus nephritis (LN). To determine the effect of CXCL13 on SLE and explore the potential mechanisms, we tested serum concentrations of CXCL13 in patients and healthy individuals and found that CXCL13 expression was high in SLE patients especially in LN patients. When we treated human renal mesangial cells (HRMCs)* in vitro* with recombinant human CXCL13, the cell proliferation was accelerated, which was tested by Cell Counting Kit-8 assay and flow cytometry. Western blot and immunofluorescence assay revealed that CXCL13 would lead to phosphorylation of extracellular signal-regulated kinase 1/2 (ERK1/2). However, the effect was weakened after the silence of CXCR5. The results of our study elaborated that high expression of CXCL13 could be involved in the pathogenesis of LN.

## 1. Introduction

Systemic lupus erythematosus (SLE) is a typical autoimmune disease with abundant production of autoantibodies [1]. Through the binding of antigen and antibody, the immune complexes develop and deposit in various blood vessels, leading to multiorgans or systems involvement [2, 3], which severely disturb the quality of life of patients [4, 5]. Although recent studies about the pathogenesis of SLE have achieved great progress, the exact mechanism remains ambiguous [6].

Lupus nephritis (LN) is the most common and serious complication in SLE patients characterized by proteinuria, hematuria, drop glomerular filtration rate, or renal dysfunction [7, 8]. It is often accompanied by pathological changes of podocytes [9–11], mesangial cells [12, 13], or renal interstitial [14]. Up to 20% of LN patients develop into end-stage renal disease, some even to death [15, 16]. Renal involvement deserves enough attention, because it is an important factor to assess the prognosis of SLE [17]. Therefore, it is necessary to explore the possible mechanisms of LN.

As the main segment in SLE, immunological abnormalities run through the whole process of this disease. In the past few years, B cell-attracting chemokine CXC ligand 13 protein, namely, CXCL13 or B lymphocyte chemoattractant (BLC), has been discovered in SLE [18, 19]. Researchers found that it was correlated with disease activity and renal involvement. The pathogenetic role of CXCL13 in podocytes has been found by Worthmann et al. [20]. However, there were few reports about the relationship between CXCL13 and mesangial cells.

In this study, we treated HRMCs* in vitro* with recombinant human CXCL13. The results revealed that CXCL13 promoted the proliferation of HRMCs by activating ERK1/2-MAPK signaling pathway. Interestingly, the effect became noteless when CXCR5, receptor of CXCL13, was silenced. Therefore, our data suggested that CXCL13 in association with CXCR5 played an important role in LN and might be a new target in LN treatment.

## 2. Materials and Methods

### 2.1. Patients and Controls

SLE patients were randomly recruited from Department of Rheumatology, Affiliated Hospital of Nantong University, from June 2013 to December 2014. All patients were diagnosed according to the American College of Rheumatology Criteria for the classification of SLE [21, 22]. Those with other autoimmune diseases, severe infections, or cancers were excluded. Healthy controls were from the Center of Health Examination of the same hospital. The study was approved by the Ethics Committees. Informed consent was obtained from all subjects.

### 2.2. Cells

Human renal mesangial cells (HRMCs) were purchased from JENNIO Biological Technology and cultured in RPMI-1640 medium (HyClone) with 10% fetal bovine serum (FBS, Gibco) at 37°C in 5% CO_2_. Cells were treated with recombinant human CXCL13 (R&D Systems, Catalog Number 801-CX-025).

### 2.3. ELISA

The concentration of CXCL13 in the serum of SLE patients and healthy controls was measured by ELISA using a Human CXCL13/BLC/BCA-1 Immunoassay (R&D Systems, Catalog Number DCX130) according to the manufacturer's directions.

### 2.4. Transfection

Cells were seeded to a 6-well plate and incubated at 37°C, 5% CO_2_ in the incubator before transfection. When the cells covered 50% to 70% of the plate, 250 *μ*L base medium containing 100 pmol CXCR5 siRNA (Biomics Biotech, China) and 250 *μ*L base medium containing 5 *μ*L Lipofectamine® 2000 (Invitrogen by life technologies, USA) were mixed for 20 minutes. The transfection complex was added with another 1500 *μ*L base medium to each well. After 4 to 6 hours, RPMI-1640 containing 10% FBS was replaced and transfection efficiency was detected within 48 to 72 hours by Western blot.

### 2.5. Cell Counting Kit-8 (CCK8) Assay

100 *μ*L cell suspension per well was prepared in 96-well plates and preincubated for 12 hours in an incubator (37°C, 5% CO_2_). After treating with or without CXCR5 siRNA transfection, the cells were incubated with or without CXCL13 at 500 pg/mL for 4, 8, 12, or 24 hours. 10 *μ*L CCK-8 solution (Sangon Biotech, Shanghai, China) and 90 *μ*L medium were added to each well and incubated away from light for 2.5 hours. The optical density of each well was determined by a microplate reader set to 450 nm.

### 2.6. Flow Cytometry Analysis

To determine the difference of cell cycle in HRMCs with or without CXCL13 treatment, we used the flow cytometry analysis. Cells were trypsinized and centrifuged at 1000 rpm for 5 minutes, washed, and resuspended with cold PBS for three times. Then they were fixed with 70% ethanol in −20°C for 24 hours, followed by centrifuging, washing, resuspending in 500 *μ*L PBS, and permeabilizing with PBS containing 1% Triton X-100. After 10 minutes, RNaseA was added and the cells were stained with 200 *μ*L PI (0.05 mg/mL) for another 20 minutes in the dark. The fluorescence of PI was measured by Flow Cytometer. Cell proportion was calculated by MFLT32 Soft. Cell proliferation status was evaluated mainly by the ratio of S phase.

### 2.7. Western Blot

Cell proteins were extracted with RIPA Lysis Buffer (Beyotime Biotechnology, China) according to the manufacturer's instructions. With 10% sodium dodecyl sulphate-polyacrylamide gel electrophoresis (SDS-PAGE), the proteins were transferred to PVDF membranes. After blocking with 5% skimmed milk in TBS, membranes were incubated with primary antibodies for anti-CXCR5 (Abcam, EPR8837), phospho-ERK, total ERK (Santa Cruz, USA), and GAPDH overnight at 4°C. Followed by washing, blots were incubated with horseradish peroxidase-labelled goat anti-rabbit IgG (NeoBioscience, ANR02-1) as the secondary antibodies for one hour. The signal was detected by ECL.

### 2.8. Immunofluorescence

Cells were seated to the slides of cells in 24-well plates and incubated overnight before treatment. The supernatant was discarded. Cells were washed with ice-cold PBS and fixed with 4% paraformaldehyde for 40 minutes at room temperature, then blocked with Immunol Staining Blocking Buffer (Beyotime Biotechnology, China) followed by washing with PBS for three times, and incubated with anti-CXCR5 (Abcam, EPR8837) or anti-pERK antibody (Santa Cruz, CA, USA) as primary antibody overnight and Dylight 594, goat anti-rabbit IgG as secondary antibody for one hour in dark. Nuclei were stained with Hoechst (Beyotime Biotechnology, China). The slides were turned over with Antifade Mounting Medium (Beyotime Biotechnology, China). Images were acquired using a fluorescent microscope.

### 2.9. Statistical Analysis

All experiments were repeated three times at least. Normally distributed data were presented as mean ± standard deviation (SD) and skewed data as the median (interquartile range, IQR). Mann–Whitney rank sum test, Chi-square analysis, Student's* t*-test (2 groups), and one-way analysis of variance (ANOVA) (more than 2 groups) were used to indicate the differences with SPSS 20.0. Spearman's test was used to assess the correlation of CXCL13 with SLEDAI or renal SLEDAI score. The results were plotted with SigmaPlot 10.0. *P* < 0.05 was defined as being significant.

## 3. Results

### 3.1. The Expression of CXCL13 Was Increased in SLE Especially in LN

**Figure 1:**
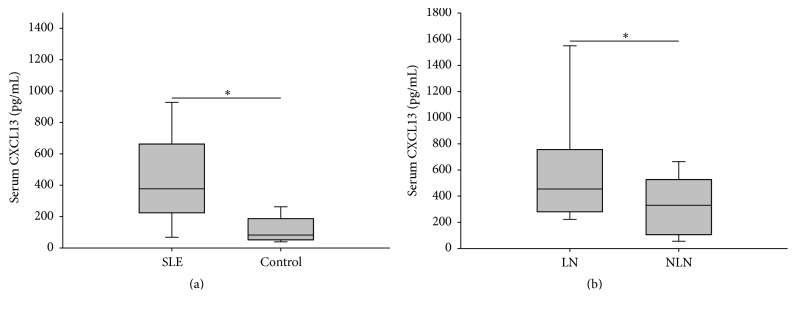
The expression of CXCL13 was increased in SLE especially in LN. (a) Serum CXCL13 of SLE patients and healthy controls. ^*∗*^ *P* < 0.001. (b) Serum CXCL13 of lupus nephritis (LN) and nonlupus nephritis (NLN). ^*∗*^ *P* = 0.004.

The clinical characteristics of subjects with SLE and controls were shown in Table 1. Of the 70 patients, there were 34 with renal involvement (LN) and 36 without (NLN). SLEDAI score and renal SLEDAI (rSLEDAI) score were assessed [23]. LN was defined as persistent proteinuria > 0.5 grams per day or more than 3+ on urine dipstick testing or cellular casts (maybe red cell, hemoglobin, granular, tubular, or mixed). These patients had active LN when we investigated. 32 healthy controls were matched by age and gender. Previous studies had reported that the expression of CXCL13 increased in SLE [19, 24]. Similarly, our results by ELISA indicated that the serum concentrations of CXCL13 in patients were higher than that of the healthy controls [376.92 (223.85–661.94) versus 82.46 (52.33–187.56) pg/mL, *P* < 0.001]. Particularly, the concentrations of patients with LN were even higher than those without [455.06 (280.04–756.84) versus 329.81 (105.42–526.98) pg/mL, *P* = 0.004] (Figure 1). Besides, serum CXCL13 concentration was positively correlated with SLEDAI score (*r* = 0.258, *P* = 0.03) and significantly positively correlated with rSLEDAI (*r* = 0.748, *P* < 0.001, data not shown).

### 3.2. CXCL13 Promoted the Proliferation of HRMCs

**Figure 2:**
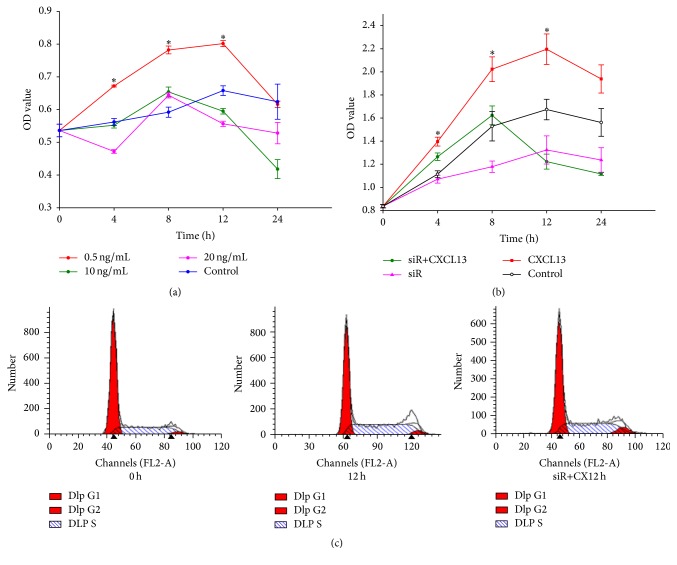
CXCL13 promoted the proliferation of HRMCs. (a) Cells were treated with CXCL13 at 0.5 ng/mL, 10 ng/mL, and 20 ng/mL. 0.5 ng/mL (500 pg/mL) was optimal concentration. (b) With 500 pg/mL CXCL13, the proliferation ability of HRMCs was shown. Both *P* values of CXCL13 versus control and siR + CXCL13 versus CXCL13 were less than 0.05. (c) Cell cycles of HRMCs with CXCL13 for 0 h and 12 h and transfected cells for 12 h were shown. Results indicated that CXCL13 promoted cells to enter S phase. However, the effect was weakened after the silence of CXCR5. ^*∗*^ *P* < 0.001.

To investigate whether CXCL13 promoted the proliferation of HRMCs so as to be involved in SLE progression, we treated HRMCs with CXCL13 at 0.5 ng/mL, 10 ng/mL, and 20 ng/mL. Results were shown in Figure 2(a). The concentration 0.5 ng/mL (500 pg/mL) was optimal in our preliminary experiment which was also adopted by Worthmann et al. [20]. The proliferation ability of HRMCs was stimulated by 500 pg/mL CXCL13 determined by CCK8 (Figure 2(b)) and FCM (Figure 2(c)). Following CXCL13 treatment, the cell proliferation status was significantly improved. The ratio of cells in S phase in the treatment group was higher than that in the control group [(49.76 ± 1.11)% versus (34.96 ± 0.08)%, *P* < 0.001] and G1 phase decreased from (61.90 ± 0.29)% to (47.42 ± 0.71)%, *P* < 0.001.

### 3.3. HRMCs Responded Mildly to CXCL13 after the Silence of CXCR5

**Figure 3:**
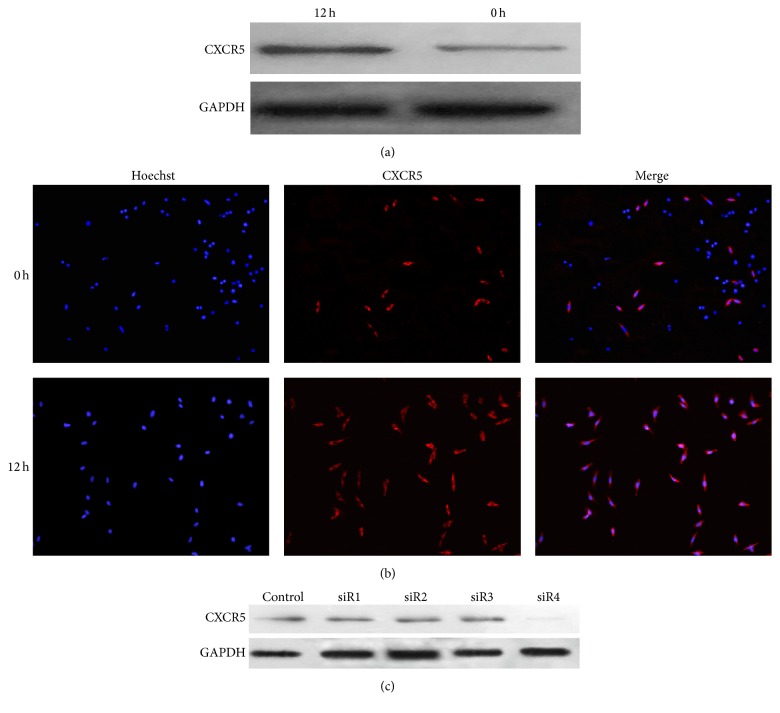
Positive expression of CXCR5 in HRMCs. (a and b) Western blot and immunofluorescence showed CXCR5 expression whether under stimulation of CXCL13 or not. (c) Transfection by siR4 was efficient.

CXCR5 was reported to be the unique receptor of CXCL13 [25]. We found a positive expression of CXCR5 in HRMCs. After stimulation with CXCL13, the expression of CXCR5 increased (Figures 3(a) and 3(b)). Then we used transfection technology to silence CXCR5; Western blot indicated that siR4 was efficient (Figure 3(c)). To investigate whether CXCR5 was involved in proliferation of HRMCs, we repeated CCK8 and FCM to test cell proliferation status after the silence of CXCR5. Results presented slight proliferation of transfected cells treated with CXCL13 compared to normal cells without any treatment (Figures 2(b) and 2(c)). The quantified results of S phase from three independent experiments were shown in Table 2.

### 3.4. CXCL13 Triggered ERK Tyrosine Phosphorylation

**Figure 4:**
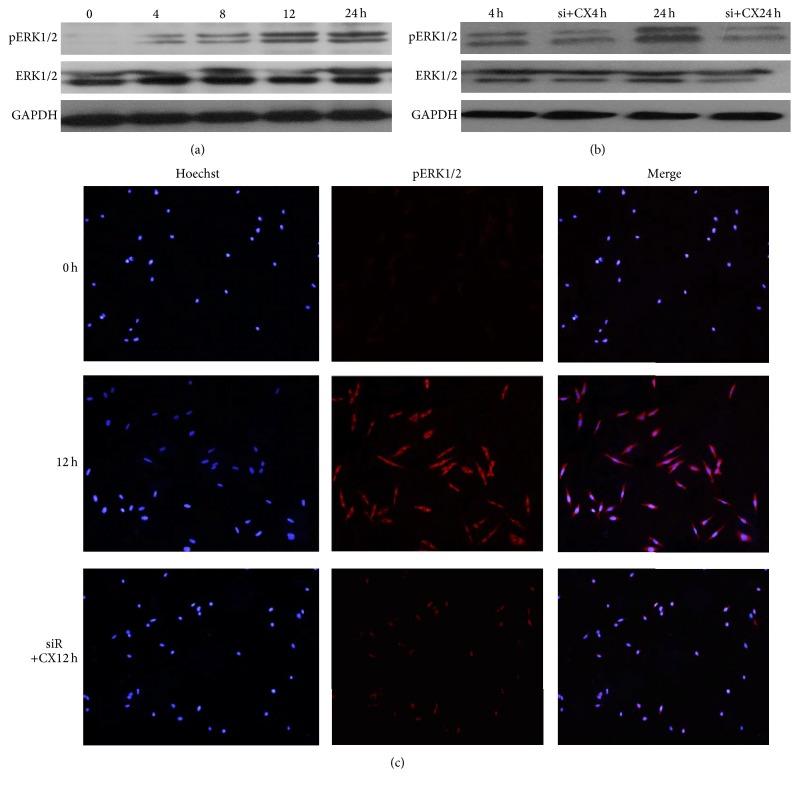
CXCL13 triggered ERK tyrosine phosphorylation. (a) Upon 4 hours, CXCL13 triggered ERK tyrosine phosphorylation of HRMCs as time went on. (b) ERK tyrosine phosphorylation decreased in CXCL13 treatment after the silence of CXCR5 compared to CXCL13 treatment alone. (c) The same result with (a) and (b) was found by immunofluorescence. The top row was detected at 0 hours and the middle at 12 hours after CXCL13 treatment. The bottom presented pERK of cells treating with CXCL13 for 12 hours after transfection.

The extracellular signal-regulated kinase (ERK, including ERK1 and ERK2) was a crucial member of mitogen-activated protein kinase (MAPK) family [26]. ERK phosphorylation (pERK) was required for cell proliferation and differentiation [27, 28]. Previous research had reported the connection of ERK1/2 and cell proliferation [29]. We tried to find out possible mechanism involved in HRMCs. Our results indicated that CXCL13 triggered ERK tyrosine phosphorylation in HRMCs in comparison with the control group. In CXCR5 silenced cells, the phosphorylation was reversed. Western blot and immunofluorescence experiments were performed to recognize pERK1/2 (Figure 4). These data were in line with the results above.

## 4. Discussion

CXCL13 is a member of chemokine superfamily and the main function is effectively chemoattracting B cells. It is produced mainly by dendritic cells in secondary lymphoid organs and can induce generation of secondary lymphoid tissue in peripheral organs [19]. CXCL13 plays an important role in the formation of germinal centers, while germinal center is required in B lymphocytes development and maturation [30, 31]. As a result, it is an important element in autoimmune microenvironment. In 2001, Ishikawa et al. discovered high expression of CXCL13 in kidney of elderly BWF1 mice [24]. After that, Schiffer et al. demonstrated that serum levels of CXCL13 in SLE patients were higher than those of healthy controls and the levels of patients with LN were even higher than those without. They discovered CXCL13 in inflammatory infiltrates of nephritic NZB/W-F1 mice.* In vitro*, CXCL13 induced production of proinflammatory factors (CXCL1, CXCL12, MIF, and LIF) in human podocytes, which indicated that CXCL13 was closely related to renal inflammation [19, 20]. Recently, CXCL13 has been reported to participate in SLE-related autoimmune hemolytic anemia [32]. We also found that the expression of CXCL13 was high in Chinese SLE patients especially in LN patients and it was positively correlated with SLEDAI especially with rSLEDAI. According to these results, we hypothesize that CXCL13 may increase more when complications occur in SLE.

The receptor of CXCL13 is CXCR5, 7-transmembrane G protein-coupled receptors, mainly expressed in mature B cells and follicular helper T cells [25]. Research has reported that CXCL13-CXCR5 axis plays an important role in immune responses [33–35]. Moreover, it has been reported that CXCL13 mediated cell proliferation [36]. In our study, CXCL13 accelerated HRMCs proliferation, which was mainly presented by promoting cells to enter S phase followed by the activation of ERK1/2. Interestingly, when we silenced the expression of CXCR5, the proliferation was weakened. As HRMCs proliferation was involved in pathogenesis of LN [37, 38], our study highlighted the significant role of CXCL13-CXCR5 axis in HRMCs proliferation and further explained the importance of CXCL13 in LN.

In summary, our results point out that CXCL13 expression is high in LN. It may promote the proliferation of mesangial cells by combination with CXCR5 via ERK1/2 pathway to be involved in pathogenesis of LN. Blocking off CXCL13-CXCR5 axis is expected to become a new therapeutic strategy targeting LN.

## Figures and Tables

**Table 1:** Clinical characteristics of patients with SLE and healthy controls.

	SLE	Controls	*P*	LN	NLN	*P*
Number	70	32	—	34	36	—
Sex (female/male)	63/7	29/3	0.8	33/1	30/6	0.1
Age (years)	36.8 ± 12.4	35.4 ± 7.9	0.5	38.8 ± 12.6	34.9 ± 12.1	0.2
SLEDAI score	13.00 ± 5.79	—	—	15.41 ± 5.32	11.03 ± 5.56	0.001

SLEDAI: systemic lupus erythematosus disease activity index.

**Table 2:** Ratio of cells in S phase from different groups.

	S phase (mean ± SD)	*P*
Control%	34.96 ± 0.08	−
CXCL13%	49.76 ± 1.11	<0.001 (versus control%)
siR + CXCL13%	40.10 ± 0.46	<0.001 (versus CXCL13%)

Statistical analyses were performed by ANOVA followed by Dunnett's test.
